# Efficacy of the Probiotic *L. brevis* in Counteracting the Demineralizing Process of the Tooth Enamel Surface: Results from an In Vitro Study

**DOI:** 10.3390/biom14050605

**Published:** 2024-05-20

**Authors:** Serena Altamura, Francesca Rosaria Augello, Eleonora Ortu, Davide Pietropaoli, Benedetta Cinque, Mario Giannoni, Francesca Lombardi

**Affiliations:** 1 Department of Life, Health & Environmental Sciences, University of L’Aquila, Building Rita Levi Montalcini, Coppito, 67100 L’Aquila, Italy; serena.altamura@graduate.univaq.it (S.A.); francescarosaria.augello@univaq.it (F.R.A.); eleonora.ortu@univaq.it (E.O.); davide.pietropaoli@univaq.it (D.P.); francesca.lombardi@univaq.it (F.L.); 2 Ph.D. School in Medicine and Public Health, University of L’Aquila, Building Rita Levi Montalcini, Coppito, 67100 L’Aquila, Italy; 3 Center of Oral Diseases, Prevention and Translational Research—Dental Clinic, 67100 L’Aquila, Italy; 4 Oral Diseases and Systemic Interactions Study Group (ODISSY Group), 67100 L’Aquila, Italy

**Keywords:** dental enamel, demineralization, probiotic *Lactobacillus brevis*, scanning electron microscopy/energy-dispersive X-ray spectroscopy, mineral composition

## Abstract

Background. Enamel plays an essential role in protecting the underlying layers of the human tooth; therefore, preserving it is vital. This experimental study aimed to evaluate the potential ability of *L. brevis* to counteract the action of a demineralizing agent on dental enamel morphology and mineral composition in vitro. Methods. The sample consisted of 12 healthy human posterior teeth. The coronal portion of each tooth was subdivided into two equal parts longitudinally. The specimens were randomly divided into four groups: artificial saliva, *L. brevis* suspension, demineralizing agent (DA), and DA plus *L. brevis*. Scanning electron microscopy (SEM) and energy-dispersive X-ray spectroscopy (EDS) were used to evaluate the surface micromorphology and the mineral content, respectively. The statistical analysis was conducted using a one-way ANOVA, followed by Tukey’s post hoc test. Results. SEM analysis did not highlight significant changes in the enamel microstructure of *L. brevis*-treated specimens compared to the control. DA-induced damage to the enamel structure was drastically reduced when the specimens were contextually exposed to the probiotic. The treatment with DA substantially reduced the weight % of crucial enamel minerals, i.e., Ca and P. Notably, the probiotic was able to reverse the demineralization process, bringing Ca and P weight % back to basal levels, including the Ca/P ratio. Conclusions. The findings indicate that *L. brevis* is able to efficiently protect the dental enamel surface from the damage caused by DA and increase the enamel resistance to demineralization. Overall, *L. brevis* confirms its efficacy in preventing or counteracting the action of carious lesions through a novel mechanism that protects the tooth surface under a chemical challenge that mimics the caries process.

## 1. Introduction

Dental caries is a prevalent, complex, chronic illness arising from an ecological imbalance in the oral microbiota and mainly relies on forming a pathogenic biofilm [1,2]. In addition, the oral cariogenic pathogens result in demineralization of enamel hydroxyapatite crystals and proteolytic breakdown of the structure of tooth hard tissues [3]. Dental enamel possesses exceptional qualities that shield the inner layers of teeth from heat, mechanical stress, discoloration, and the entrance of germs and other microbes, as well as protecting the underlying dentin layer from outside harm [4,5].

It has been shown that some probiotics suppress the growth of cariogenic bacteria colonies, release antibacterial compounds, outcompete oral pathogens for nutrition, and lessen the production of biofilms [6,7]. According to one definition, probiotics are “live microorganisms which, when administered in adequate amounts, confer a health benefit to the host”. With modes of action including adhesion, coaggregation, and competitive inhibition, as well as the ability to bolster the mucosal barrier and influence the immune system to have an anti-inflammatory effect, they are effective in both the prevention and treatment of a wide variety of disorders [8,9,10,11,12,13,14,15].

Taking some probiotics might be an excellent way to maintain dental health, acting as a substitute or supplementary treatment for mouth disorders [16]. Probiotics, particularly Lactobacillus, have been shown to balance the microbiota in the mouth, alter immunological and inflammatory responses, secrete antibacterial compounds, outcompete oral pathogens for nutrients, fortify the mucosal barrier, lessen the formation of pathogen biofilms, and either prevent or mitigate periodontal disease [6,17,18].

*Lactobacillus brevis CD2*, recently reclassified as *Levilactobacillus brevis (L. brevis)* [19], is a multifunctional probiotic used mainly in oral medicines, having been evaluated in various conditions, including caries [20,21], gingivitis and periodontitis [22,23,24,25,26], oral mucositis [27,28], Behçet’s disease, and aphthous oral ulcers [29,30]. Other than antagonizing pathogenic bacteria, *L. brevis* has been shown to influence the host inflammatory response by downregulating the inducible nitric oxide synthase 2 (NOS2) pathway, thus inhibiting the production of NO [21,22], a potent inflammatory mediator. Most beneficial effects on the oral cavity after *L. brevis* assumption, both in healthy and disease states, have been attributed to a high level of arginine deiminase (ADI) [21,22,31], one of its most exciting peculiarities. ADI, lacking in human cells, is an enzyme that converts arginine present in the oral cavity, leading to the formation of citrulline and ammonia (NH_3_). In an aqueous solution, NH_3_ forms ammonium ions (NH_4_^+^), which are crucial to maintaining the physiological salivary pH and thus preventing enamel decalcification [32,33]. Indeed, levels of salivary NH_3_ were found to be upregulated in dental caries-free, healthy human subjects compared to caries-active ones [33,34,35]. These differences could be attributed to higher bacterial ADI levels. Given that NH_3_ is a proton acceptor (i.e., a weak Lewis base), the higher pH and acid-neutralizing conditions generated create an unfavorable environment for the growth and preponderance of cariogenic microorganisms. Thus, the alkaline-producing capacity of *L. brevis* may be a great advantage of this probiotic when considering oral application. These characteristics make *L. brevis* a candidate to control the development and progress of oral disorders. By metabolizing arginine, for which it has a higher chemical affinity, ADI prevents the use of the latter by both arginase and NOS2, thus inhibiting, respectively, the synthesis of polyamines (i.e., putrescine and spermidine) involved in halitosis and tumor cell proliferation and NO, one of the most potent mediators of inflammation that is also associated with periodontal inflammation [22,31].

However, despite the numerous and growing literature supporting the efficacy of *L. brevis* in oral medicine, there is a current need for sufficiently valid evidence concerning the impact of this probiotic on enamel demineralization. Therefore, the present research aimed to verify in vitro whether *L. brevis* can counteract the damage caused by a demineralizing agent on the surface enamel. Scanning electron microscopy (SEM) was utilized to assess the morphology of the surface enamel. Energy-dispersive X-ray spectroscopy (EDS) was employed to examine the impact of *L. brevis* suspension on the demineralization of enamel and the elemental composition of the tooth surface, with particular attention to modifications in the composition of Ca and P. Combining these analytical techniques gives a thorough picture of the morphological and chemical changes to the tooth surface.

## 2. Materials and Methods

### 2.1. Tooth Collection and Selection and Specimen Preparation

Sixteen human teeth extracted for orthodontic purposes were collected from subjects aged between 20 and 40 in the Dental Clinic of the University of L’Aquila after obtaining the donors’ informed consent under a protocol approved by the Internal Review Board of the University of L’Aquila, n. 48/2022, 22 November 2022. After careful clinical evaluation and diagnosis, selective extractions (premolars and molars) were planned and performed by the dental clinician for seven healthy patients who, due to severe dental overcrowding (lack of space in the arches), protrusion of the anterior teeth, and bite correction, had to undergo orthodontic therapy. Teeth with caries, cracks, or defects were excluded. Twelve of the sixteen teeth extracted were completely healthy and free of cavities, cracks, and similar structural defects and were finally retained for the study. The teeth were disinfected using 0.5% chloramine, stored in distilled water, and scaled with Gracey curettes (LM Dental, Parainen, Finland) and machine-driven ultrasonic scalers (LM Dental, Parainen, Finland) to remove all tissue tags adhering to the tooth. Before treatment, the selected teeth were stored in saline at 4 °C, adding 0.1% thymol solution (pH 7.0) to prevent the growth of bacteria until use. All the teeth were subsequently sectioned at the cementoenamel junction in a mesiodistal direction using a low-speed micromotor and diamond disc bur to separate the crown from the root portion. Each crown surface was then subdivided into two sections. The 24 specimens were randomly assigned to each treatment group (n = 6 per group): control, probiotic, demineralizing agent (DA), and DA plus probiotic.

### 2.2. Preparation of the Artificial Saliva

The artificial saliva solution was freshly prepared before the tests and contained sodium chloride (NaCl) [0.4 g/L], potassium chloride (KCl) [0.4 g/L], sodium biphosphate (NaH_2_PO_4_·2H_2_O) [0.69 g/L], calcium chloride dihydrate (CaCl_2_·2H_2_O) [0.9 g/L], sodium sulfide nonahydrate (Na_2_S·9H_2_O) [0.005 g/L], and urea [1 g/L]. To simulate the basal concentration of salivary glucose, the artificial saliva was supplemented with glucose [0.01 g/L]. The artificial saliva solution components were obtained from Sigma Aldrich (St. Louis, MO, USA). The pH of the solution was checked using a digital pH meter (Mettler Toledo, Columbus, OH, USA) and adjusted to a neutral pH of 7.0 by adding 1N sodium hydroxide (NaOH).

### 2.3. Preparation of L. brevis Suspension

The probiotic suspension was prepared by first dissolving one Mucomixx lozenge (Mendes Srl, Ardea, Italy); one lozenge contained 10^9^ CFU of *Levilactobacillus brevis* DSM27961/CNCM I-5566 in 10 mL of phosphate-buffered saline (PBS, EuroClone, West York, UK). The suspension was centrifuged at 8600× *g*, washed twice in PBS, and resuspended in 4 mL of artificial saliva. *L. brevis* suspension was prepared for the in vitro probiotic treatment to obtain a final 16.6 × 10^6^ CFU/mL concentration.

### 2.4. Preparation of the Demineralizing Solution

Citric acid (Sigma Aldrich, St. Louis, MO, USA) at 6% in ultrapure H_2_O (pH 2.2) was used as a demineralizing solution.

### 2.5. In Vitro Treatments

The study on the enamel surfaces of all specimens was conducted by applying a pH-cycling model mimicking the oral environment.

All 24 specimens were carefully placed in a sterile 12-well polystyrene cell culture plate (Corning Incorporated, New York, NY, USA), immersed in 4 mL of the respective solution, and incubated in a thermomixer (Eppendorf, Hamburg, Germany) under continuous stirring at 37 °C for five days, ensuring optimal conditions for the experiment.

The 24 sections were rigorously and randomly assigned to each treatment group (six each) as follows:

- Group 1: The specimens were immersed in 4 mL of artificial saliva, which was replaced daily with fresh artificial saliva. This protocol was repeated each day for five days.
- Group 2: The specimens were treated with the probiotic solution at 16.6 × 10^6^ CFU/mL for 6 h/day, after which they were extensively washed and incubated in fresh artificial saliva for another 18 h. This protocol was repeated each day for five days.
- Group 3: The specimens were immersed for 2 min in the DA (6% citric acid), after which they were extensively washed and incubated in fresh artificial saliva for 24 h. This protocol was repeated each day for five days.
- Group 4: The specimens were immersed for 2 min in the DA (6% citric acid) and then in probiotic suspension (16.6 × 10^6^ CFU/mL) for 6 h, after which they were extensively washed and incubated in artificial saliva for another 18 h. This protocol was repeated each day for five days.

The pH at all conditions was subsequently recorded by pipetting the solution from each well at baseline, 3, 6, 12, and 24 h using a pH meter (Mettler Toledo, Columbus, Ohio, OH, USA). The mean descriptive of all the baseline and post-pH cycle regime measurements were conducted, and no significant change from baseline values was recorded (pH range: 6.95–7.03). After final exposure to the treatments, all the specimens were washed four times (5 min each) with distilled water and then dehydrated in ethanol solutions (30–50–70–90%) and two times in 100% ethanol for 10 min each.

### 2.6. Scanning Electron Microscopy (SEM) and Energy-Dispersive X-ray Spectroscopy (EDS)

SEM observations were carried out by a Zeiss FEG SEM Gemini SEM 500 (Microscopy Centre, University of L’Aquila). The SEM was operated at 10 kV and an 8.5 mm working distance under environmental conditions and variable pressure (23 Pascals), without metalizing the specimens to avoid artifacts and cracks. Scanning electron micrographs of the enamel surface were obtained randomly with magnifications of 500×, 2000×, 5000×, and 10,000×. The obtained micrographs were evaluated descriptively, observing the variations in the micromorphology of the dental enamel of all the different analyzed groups.

The qualitative elemental analysis of the specimens was carried out by EDS, performed by the SEM equipped with microanalysis Oxford EDS (Oxford Aztec Live with Detector Ultim Max 100). The operating parameters were 8.5 mm working distance, 10 kV accelerating voltage, and magnification of 500×. The enamel chemical elements’ concentrations by weight (%) were analyzed. In particular, our attention was focused on the Ca and P content and their ratio (Ca/P) to evaluate the change in the mineral density after the different treatments.

### 2.7. Statistical Analysis

The data were evaluated using GraphPad Prism version 8.02 (GraphPad Software, San Diego, CA, USA). To compare the mean values among groups, we used a one-way analysis of variance (ANOVA) followed by Tukey’s post hoc test. Data were expressed as means ± SD. The *p* values were considered statistically significant when they were lower than 0.05.

## 3. Results

### 3.1. Effect of L. brevis on the Surface Morphology of Dental Enamel

**Figure 1:**
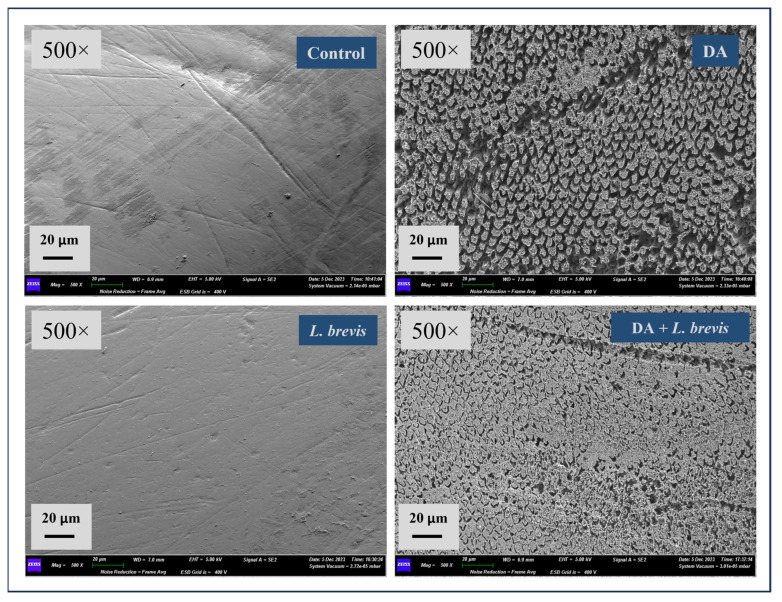
SEM representative micrographs at 500× magnification of specimens following different treatments. Control enamel, enamel after demineralized agent (DA), enamel treated with *L. brevis* suspension, and enamel treated with DA plus *L. brevis* suspension.

SEM analysis was used to investigate and compare the surface morphology of each treated specimen with that of the control. Representative SEM micrographs of the enamel surface at 500× magnification after the different treatment conditions are shown in Figure 1. Control specimens showed the typical aspect of sound enamel with a relatively smooth and flat surface in the presence of polishing scratches. The surface of the specimens treated with *L. brevis* showed no signs of enamel damage with no prismatic exposure or interprism gaps, suggesting that the physiological structure was preserved. As expected, an irregular surface pattern was observed in all the specimens exposed to DA with clear signs of loss of enamel integrity, prisms’ dissolution, exposure of enamel rods and disorganization of the interprismatic rod’s structure, and well-pronounced morphological defects with areas showing the formation of pits, pores, and micro-erosion, as evident even at 500× magnification. Of note, the specimens exposed to DA and treated with *L. brevis* revealed significantly less erosion and distortion of the enamel surface, with strongly attenuated prism exposure and notably reduced interprism gaps, as evident in the micrograph shown in Figure 1.

**Figure 2:**
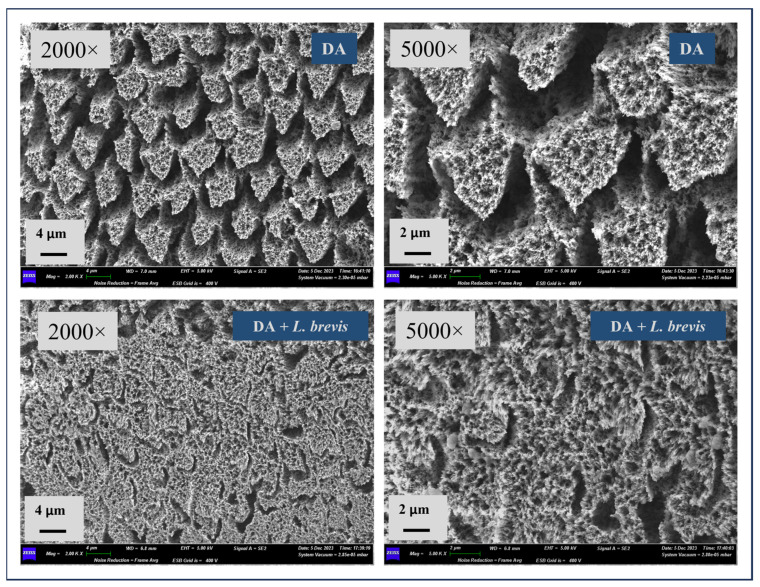
Representative SEM micrographs of specimens at 2000× and 5000× magnifications. The top micrographs (specimens treated with DA) highlight a complete loss of surface integrity, with firm prism core dissolution and loss of interprismatic rods protruding, along with damaged and fragmented enamel. At increased magnification (5000×), pits, pores, and micro-erosion were well observed. The bottom micrographs (specimens treated with DA + *L. brevis*) display a reduced dissolution of the prisms and fewer holes and eroded areas than the specimens treated with the demineralizer alone.

Figure 2 shows representative SEM images at magnifications of 2000× and 5000× for the specimens exposed to DA or a combination of DA and probiotic *L. brevis*. At these higher magnifications, the distinctive loss of enamel rods and the typical fish scale-shaped structures were even more noticeable on the specimens exposed to the DA surface, as evident from the representative images shown in Figure 2. At these higher magnifications, the probiotic effect in counteracting DA’s erosive action appeared even more appreciable. Therefore, the specimens treated with DA in combination with the probiotic confirmed a clear improvement in the enamel surface, with the formation of rod-like crystallites in certain directions of growth. The dissolution of the prism core was less present compared to demineralized specimens, and there were much more attenuated micro-erosions on the surface and significantly reduced numbers of holes and eroded areas.

### 3.2. Effect of L. brevis on the Elemental Composition of Dental Enamel

**Figure 3:**
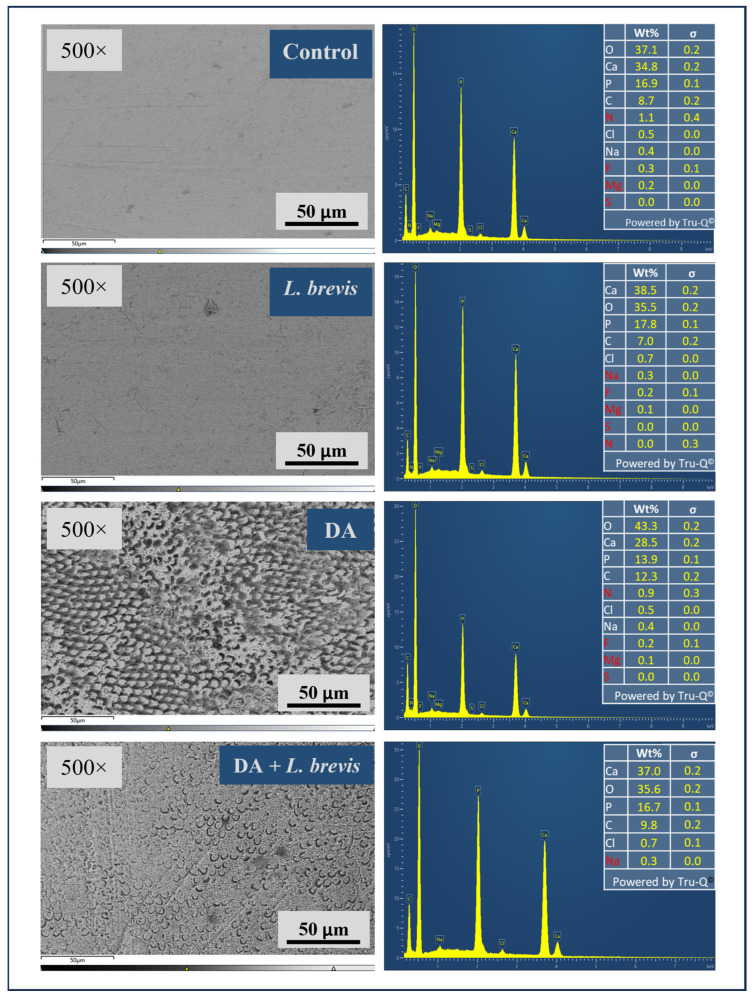
Representative SEM images of the enamel surface for each group (left panels, from top to bottom: control, *L. brevis*, DA, and DA + *L. brevis)* with relative EDS analysis (right panels).

The mineral content in weight % of the enamel surface of each specimen in the four groups was measured by means of EDS, a microanalytical technique widely used in conjunction with SEM to evaluate structural and morphological variation and quantitatively estimate the amounts of elemental composition. Figure 3 shows representative SEM images and relative EDS analysis of specimens from the four treatment groups: control, DA, *L. brevis*, and DA + *L. brevis*. All SEM microphotographs (magnification 500×) confirmed the observations reported above. Therefore, control or probiotic-treated specimens showed normal and regularly smooth enamel surfaces (Figure 3, images on the left). On the other hand, after five citric acid exposures (DA treatment), the SEM micrographs confirmed the typical picture of a demineralized enamel with interprismatic holes. Moreover, imaging revealed an evident improvement in the enamel ultrastructure of the specimens treated with DA plus *L. brevis* compared to DA alone, with a smoother enamel surface and a decreased number and size of pores. The detections of the inorganic compounds on the enamel surface are shown in the right panels of Figure 3 as EDS spectra, and the corresponding values (weight %, σ) are recorded in the tables. While treatment with *L. brevis* did not seem to significantly affect the elemental composition of the enamel compared to the control, as evidenced by the representative case shown in Figure 3, the surface weight % of Ca, P, C, and O were significantly affected by demineralization treatment. In particular, the results indicated an evident drop in the weight % of Ca and P following the demineralization process compared to the control specimen. Of note, the surface mineral composition (Ca, O, P, and C) of specimens treated with DA plus *L. brevis* showed an elemental composition comparable to that of control or probiotic-treated samples, thus confirming the neutralizing effect of *L. brevis* against the erosive and demineralizing action of citric acid.

**Figure 4:**
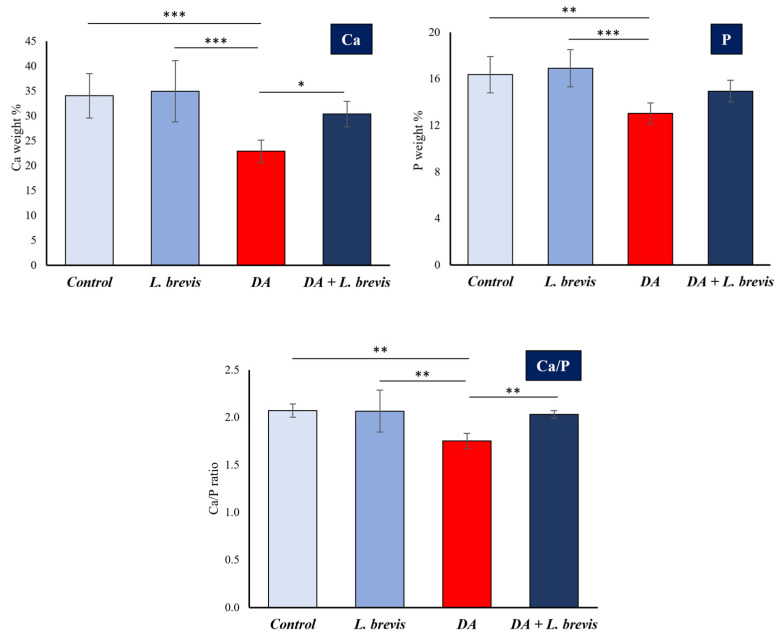
Variations of the concentration values by weight (%) of calcium (Ca) and phosphorous (P) of dental enamel analyzed using EDS in specimens from the four different groups: control, *L. brevis*, DA, and DA + *L. brevis*. The results are expressed as mean values ± SD (top panels). The values of the Ca/P ratios according to the different treatment groups are shown in the bottom histogram. Asterisks indicate statistical differences among groups (* *p* < 0.05; ** *p* < 0.01; *** *p* < 0.001).

Figure 4 reports the results expressed as means ± SD of Ca and P (weight %) at the enamel surface of specimens of the different treatment groups (control, *L. brevis*, DA, and DA + *L. brevis)*. The Ca/P ratios are also shown in the bottom histogram. It is evident that adding *L. brevis* restored the strongly reduced mean values of Ca (weight %) and Ca/P ratio in the specimens treated with the demineralizer to levels not significantly different from those of the control or probiotic alone. P levels also showed an increasing trend in specimens exposed to DA + probiotic compared to those treated with DA alone, although they did not reach statistical significance.

## 4. Discussion

Dental enamel is the most durable tissue in the body, composed of both a mineral phase and an organic component [36,37]. The mineral phase is the predominant component, accounting for 95–96% of the total weight and is mainly composed of calcium phosphate salts in the shape of massive hexagonal hydroxyapatite crystals. The other components include calcium carbonate, calcium fluoride, magnesium phosphate, and traces of other different salts [37]. The main function of the enamel is to protect the dentin layer underneath it, which is responsible for enclosing the pulp, a highly vascularized and innervated soft tissue. Because of these characteristics, enamel shields the inside of the teeth from heat, mechanical damage, discoloration, and the introduction of germs and other microbes onto the teeth. Due to its high mineral concentration, enamel is sensitive to demineralization, which is the process that ultimately results in dental caries. Initial enamel lesions can develop extremely quickly, and the cycles of demineralization and remineralization in the oral environment are in a state of equilibrium. Dental cavitation may develop if preventative measures are not taken to halt the advancement of enamel surface lesions. Therefore, the primary objective is to modulate the mineral balance in the oral cavity in a manner favorable to the remineralization of teeth.

A growing body of data in vitro and in vivo supports the use of the probiotic *L. brevis* as an appropriate therapeutic adjuvant or replacement approach in oral medicine. *L. brevis* is an obligate heterofermentative lactic acid bacterium (LAB) with many composite oral probiotic properties underlying its clinical applications (inhibition or exclusion of oral pathogens, inhibition or disruption of pathogen biofilm, persistence in the oral cavity, and anti-inflammatory effects) [20,21,22,23,24,25,26,27,28,29,30]. However, according to our knowledge, there is no specific evidence of *L. brevis*’s ability to prevent or counteract tooth enamel demineralization. This in vitro study was conducted to investigate a new potential effect of *L. brevis* in a pH-cycling model to increase the resistance of tooth enamel against acid attack and, as a result, counteract demineralization. As mentioned above, demineralization and remineralization affect the surface’s mechanical properties, including its hardness, which is directly related to its mineral content. As evidenced by the growing number of scientific studies in this context, scanning electron microscopy–energy-dispersive X-ray spectroscopy (SEM-EDS) is considered an efficient way to assess the changes in surface structure during demineralization and the in vitro remineralization process [38,39,40,41,42,43,44,45,46,47]. Thus, SEM-EDS was used to evaluate structural and morphological changes on the enamel surface and to quantitatively estimate the amounts of mineral content in the weight percentage of enamel in each treatment group. The results indicated that the probiotic *L. brevis* had the potential to counteract the demineralizing effect of citric acid on the enamel surface, therefore enhancing both the morphology and the mineral content of the enamel. Indeed, the treatment with probiotic suspension after DA exposure significantly decreased the dissolution of the enamel prisms and interprism gaps, which improved the enamel surface that had been damaged by citric acid. This was compared to the specimens that were exposed to the DA alone. The EDS investigation of the mineral composition of the enamel surface provided evidence that corroborated the results of the scanning electron microscope (SEM). The data relating to calcium and phosphorus, whose connection is a crucial indication of the remineralization process [48], were particularly interesting. It is well known that the demineralization process results in the dissolution and release of calcium and phosphate ions from the enamel surface. This results in the loss of the enamel’s traditional ultrastructure and elemental composition [48,49]. The exposure to citric acid also resulted in the breakdown of superficial calcium hydroxyapatite in our in vitro settings. This resulted in the appearance of enamel rods and increased the intensity of backscattered light from the tooth surface. Compared to the control group and *L. brevis*-treated specimens, which showed mineralized enamel surfaces in good condition and did not exhibit any changes in their characteristics, the demineralized group showed a considerable decrease in calcium and phosphorus. This led to the enamel ultrastructure being damaged due to an increase in interprismatic voids and the conversion of individual crystallites via their dissolution. However, specimens exposed to DA and then treated with *L. brevis* showed a considerable improvement in the ratio of calcium to phosphorus compared to those subjected to DA alone.

One of the limitations of the current experimental investigation was that, even though efforts were made to simulate the oral environment, some crucial parameters, such as natural salivary composition and oral microbiota, were not considered in this study. Indeed, the model used to induce enamel lesions artificially (i.e., pH cycling) is not a result of the metabolic action of a microbial biofilm, thus excluding the cariogenic action of the latter on the tooth surface, a critical factor in the caries process. The alternating demineralization and remineralization cycle occurring in pH-cycling models, mimicking the dynamics of mineral loss and gain involved in caries formation, is accepted and broadly utilized by scientists as an appropriate alternative to animal caries testing [50]. 

First, our results showed that *L. brevis* exposure did not affect the normal dental enamel surface in terms of morphology and elemental composition. Although the role of selected LABs in treating oral diseases, including caries, is well established, their acidogenic and aciduric nature has given rise to some perplexities and doubts about the possibility that they can cause tooth demineralization and increase the risk of tooth decay [51,52]. However, our findings suggest that *L. brevis*, a weak acid producer compared to other LABs, may not pose a significant risk to oral health, mainly in terms of tooth decay [53]. This provides a new perspective on the acidogenic and aciduric nature of LABs and their potential benefits and risks. On the other hand, the observed ability of *L. brevis* to prevent or repair the damage caused by the demineralizing agent in the pH-cycling model used cannot be attributed to the recognized ability of the probiotic and its antimicrobial products to antagonize the cariogenic microorganisms and biofilm formation [20,21,54,55,56]. Analogously, the production of ammonia by *L. brevis* through ADI activity, which can positively influence the balance between remineralization and demineralization of the tooth in vivo, thus helping to prevent the emergence of a cariogenic microbiota, cannot be involved in the experimental model used in the present work. Therefore, the mechanisms underlying the observed effects, particularly in terms of the remineralizing action of dental hydroxyapatite, buffer capacity, exchange of ions in the enamel, and presence of ions that facilitate (calcium and phosphate) or not (hydrogen ions) the remineralization process [57], need to be further investigated. Moreover, even if our in vitro research may not fully translate to in vivo conditions and actual consumption effects, there is, for other oral conditions, evidence of the efficacy of the probiotic product used [20,21,22,23,24,25,26,27,28,29,30]. In this context, we highlight in particular the results of the randomized, placebo-controlled clinical trials reporting the short-term effect of *L. brevis* containing lozenges vs. placebo on some oral health parameters in children with high caries risk or type 1 diabetes, which showed a significant improvement of the examined risk factors in the probiotic group compared to the relative placebo [20,21].

Finally, this study may be limited by the type of enamel samples used, which could affect the generalizability of the results to a broader range of dental conditions (i.e., the age of the subjects). At least for the range of ages of the subjects (20–40 years) whose teeth were studied, the product works. The product’s efficacy should be evaluated on dental enamel from older groups with reduced enamel mineralization or compromised oral health conditions.

Overall, under the limitations of an in vitro investigation, the findings appear encouraging and, most importantly, show that the beneficial action of *L. brevis* on dental enamel reorganization occurs after five days. Hence, *L. brevis* emerges as a powerful remineralizing agent and confirms its efficacy in preventing or counteracting the action of carious lesions through a new mechanism of action that adds to those already highlighted by previous clinical trials.

## 5. Conclusions

The present findings support the possibility of employing the multifunctional probiotic *L. brevis* as a practical approach for inducing resistance to the enamel demineralization process, stimulating remineralization, and consequently preventing early caries. Even though additional research is needed to determine the mechanisms underlying the observed effects, the results are encouraging. Moreover, further clinical studies on this issue are recommended to assess the remineralizing abilities of *L. brevis*.

## Data Availability

The data that support the findings of this study are available from the corresponding authors upon reasonable request.
